# Improving the Properties of Gray Cast Iron by Laser Surface Modification

**DOI:** 10.3390/ma16165533

**Published:** 2023-08-09

**Authors:** Qingyi Sai, Jiale Hao, Shuwen Wang, Zhi Wang

**Affiliations:** 1 College of Energy and Power Engineering, University of Shanghai for Science and Technology, Shanghai 200093, China; saiqingyi@usst.edu.cn; 2 College of Mechanical Engineering, University of Shanghai for Science and Technology, Shanghai 200093, China

**Keywords:** laser modification, vibration and noise, residual stress, corrosion, cast iron

## Abstract

Laser surface modification is a widely used technology to improve the properties of functional surfaces. In this study, the properties of gray cast iron are modified by laser surface modification, and the influence of laser quenching on the properties of cast iron in terms of frictional vibration and noise, friction and wear, internal structure, residual stress, hardness, and corrosion resistance is investigated. The experimental results show that, after high-power laser quenching, the frictional vibrations and noise of most gray cast iron specimens are decreased, but the coefficients of friction against a bearing steel counterface are increased and more stable. The surface and sub-surface hardness of all laser-quenched cast iron specimens is significantly increased. The residual stresses on the surface of the cast iron specimens are significantly increased and changed from tensile to compressive residual stresses. Experimental modal testing results show that the modal damping ratios of the laser-treated specimens are increased significantly, although their modal frequencies are not significantly changed. In addition, through the metallographic observation, XRD (X-ray diffraction) analysis, and TEM (transmission electron microscopy) observation, it is found that the microstructures of the cast iron specimen after high-power laser modification become fine-grained, and the pearlite and ferrite in the matrix become fine martensite, which leads to the improvement of the dynamical, tribological, and chemical properties of cast iron after laser modification.

## 1. Introduction

Heat treatment involves a collection of processes aiming to improve the comprehensive properties of metallic materials by modifying their initial crystalline structure. One of the major disadvantages of conventional quenching is that the entire component undergoes phase change, but in many cases, only a specific area or surface layer is required to be hardened for some machine components, such as gears, crankshafts, and cylinder liners in the automotive industry [1,2,3]. Therefore, the development and implementation of new surface modification technologies is an important and urgent task in the mechanical engineering industry.

Many traditional surface treatment techniques have been used to improve the mechanical, physical, and chemical properties of the surfaces of metal parts, such as surface coating, diamond-like carbon (DLC) [4,5], abrasive jet machining (AJM) [6,7], micro-electrical etching (MEE) [8], etc. However, based on the fast-growing laser technology, laser surface treatment is becoming more popular in functional surface modification due to its advantages that have been widely studied in recent decades [9,10,11,12,13,14,15,16]. Laser quenching has been shown to be an effective approach to improve the wear resistance, residual stress, local hardness, and structural mechanical properties of functional materials.

Biryukov et al. [17] studied the metallographic and tribological properties of laser and monolithic hardened specimens of 65Mn steel, and the experimental results showed that the friction coefficient of steel can be significantly reduced by laser quenching treatment, the wear of steel in the working process was reduced, and the service life of the workpiece was improved. Li et al. [18] combined traditional quenching and laser quenching to improve the mechanical properties of the 1.0C–1.5Cr steel. Studies showed that double quenching increased the hardness and enhanced the wear resistance of the steel. Panfil et al. [19] investigated the mechanical properties of 42CrMo4 steel by laser quenching after gas nitriding. The positive influence of laser heat treatment on Young’s modulus and hardness was confirmed for the outer ε zone as well as for the laser-quenched diffusion zone. Yao et al. [20] used laser quenching combined with liquid nitrogen to treat titanium plates at a low temperature, and found that compared with laser quenching alone, this method not only increased the thickness of the nitrided layer to 60 μm and was more uniform, but also improved the hardness and wear resistance of titanium plates.

Kulka and Pertek [21] investigated the structure and properties of the modified layer of 15CrNi6 steel boron-carburizing followed by laser quenching treatment. It was found that boron-carburizing followed by laser quenching treatment significantly increased the hardness of the carburized layer and increased the effective hardening depth, although it had a small negative effect on the surface hardness of the functional material. Yan et al. [22] improved the mechanical properties of 30CrMnSiA steel by laser quenching after nitriding. The study found that compared with nitriding or laser quenching alone, the laser quenching treatment after plasma nitriding increased the thickness of the modified layer and improved the wear resistance of 30CrMnSiA steel. Wang et al. [23] investigated the effect of laser quenching on the surface properties of low-alloy steel after plasma nitrocarburizing. The study found that the hybrid plasma carbonitriding and laser quenching significantly increased the thickness and hardness of the modified layer, and enhanced the wear resistance of the low-alloy steel.

Maharjan et al. [24] studied the effect of laser quenching cooling rate on the hardness of steel by using water as the cooling medium. The study found that the laser energy was weakened by the cooling water. Compared with other laser quenching without water cooling, the depth of the hardened layer was smaller, but the surface hardness of the laser-quenched steel was higher. Wang et al. [25] carried out laser surface quenching treatment for a heavy-duty railroad track and studied the damage mechanism of the track after laser quenching. The results showed that the surface hardness and wear resistance of the heavy-duty railroad track had been greatly improved by laser quenching, and the wear of the track after laser quenching was reduced by about 60% compared to that of the untreated track, and the damage of the track surface changed from serious adhesive wear to abrasive wear. Hu et al. [26] investigated the effects of discrete surface laser hardening and combined ultrasonic-assisted surface plastic deformation on the surface microstructure and tensile properties of Cr-Ni-Mo alloyed steel and found that fine-grained needle-like martensite appeared in the treated specimens, increasing hardness, tensile strength, and yield strength. Similar findings were made by Radkiewicz et al. [27] in their study of the mechanical properties of manganese steel after laser quenching. Li et al. [28,29,30] studied the microstructure and wear behaviors of 40CrNiMo steel after laser quenching. The study showed that the wear mechanism of curved 40CrNiMo steel consists mainly of abrasive wear, and its hardness also increased significantly after laser quenching. Similarly, Sharp et al. [31], in their study of the microstructure of gray cast iron after laser quenching, demonstrated that gray cast iron produces martensite, austenite, dendritic crystals, and pearlite after laser quenching, thereby improving its mechanical properties. In the studies of the properties of ductile iron after laser quenching, Eduardo et al. [32] treated the surface of ductile iron by laser quenching, and it was found that the hardness of the heat-treated specimens could reach up to 1145 HV, indicating that laser quenching can improve the hardness and wear resistance of ductile iron. Chen et al. [33] found that the hardness of the laser-quenched specimens increased about 3-fold, the wear depth decreased by about 35%, and the wear mechanism changed from plowing wear to adhesive wear. The results of Samar et al. [34] showed that the microstructure of ductile iron after laser quenching consisted of acicular martensite, austenite, and graphite, and that the hardness was increased nearly 6-fold compared to the base metal.

The above literature review shows that many studies on the laser hardening of steel and its alloys have been carried out, and the mechanism of metal surface hardening and wear resistance enhancement by laser quenching is comparatively well understood. However, the effect of laser quenching on the dynamical properties of metals in terms of vibration and noise performance is still relatively unexplored. In this study, in order to improve the tribological and dynamical properties of cast iron by laser surface treatment (quenching), parametric studies were carried out to optimize the laser parameters used in the surface treatments. In addition, the effect of laser quenching on the frictional vibration and noise reduction of cast iron was investigated. In addition, various experimental studies were carried out to investigate the mechanisms of surface hardening, wear and corrosion resistance, friction stabilization, and vibration and noise reduction of laser-quenched surfaces of cast iron by means of various macro/micro-scale characterizations. 

## 2. Experiment and Methodology

### 2.1. Laser Surface Treatment

**Figure 1:**
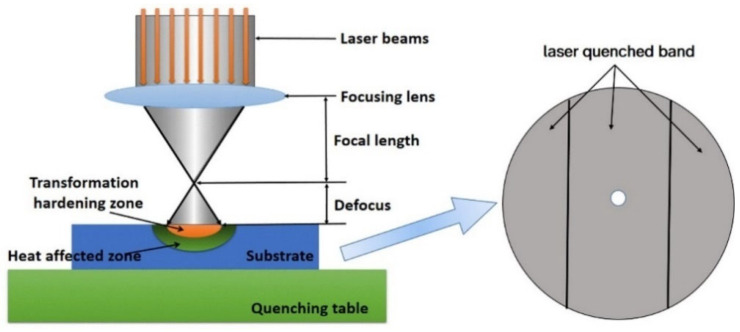
Schematic diagram of laser quenching.

Twelve gray cast iron (HT250) discs with a diameter of 70 mm and thickness of 10 mm were laser-surface-treated, which is shown in Figure 1. 

The laser surface quenching of the specimens was performed by varying the laser power and scanning speed but keeping the laser spot area constant. The specimens were treated with various LQI (Laser Quenching Index) values, defined as the ratio of laser power to the product of scanning speed and spot area. The LQI values used in this study are listed in Table 1.

### 2.2. Experimental Setup

#### 2.2.1. Tribological Tests

The UMT-TriboLab multifunctional friction and wear tester was employed to test the tribological properties of the cast iron samples. In the tribological tests, the upper specimen is a stationary GCr15 bearing steel (52100 steel) ball, while the lower specimen is a rotating cast iron disc. In all the tribological tests, the operational parameters were kept constant, i.e., normal load 20 N and relative sliding speed 1 m/s, and all tests were carried out under dry conditions. 

#### 2.2.2. Vibrations and Noise

During the tribological tests with the UMT-TriboLab multifunctional tribological tester manufactured by Bruker Technology Co., Ltd., Billerica, MA, USA, the frictional vibrations and nose were measured by a triaxial accelerometer (Kistler 8766A50M5) made by Kistler China Ltd., Shanghai, China and a sound pressure sensor (INV9206, BOIVN, Beijing, China) via the DASP (data acquisition and signal processing) system developed by Beijing Oriental Institute of Vibration and Noise (BOIVN).

## 3. Results and Discussion

### 3.1. Coefficients of Friction

**Figure 2:**
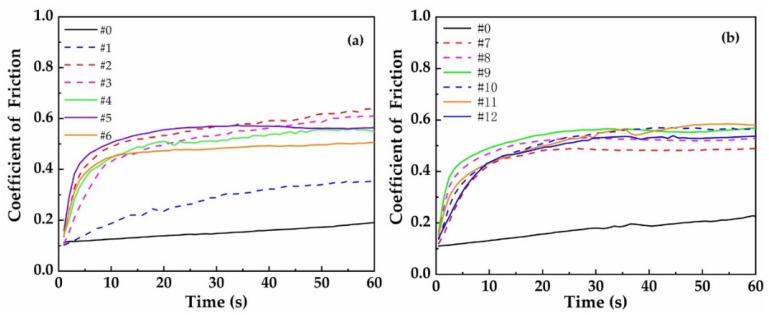
Coefficients of friction between a bearing steel ball and cast iron disc: (**a**) untreated specimen and Groups I and II (Nos. 0–6); (**b**) untreated specimen and Groups III and IV (Nos. 0, 7–12).

Figure 2 shows the coefficients of friction (COFs) of the dry sliding contacts between a bearing steel ball and a cast iron disc with or without laser quenching, under a normal load of 20 N and sliding speed of 1 m/s in a sliding distance of 60 m. Figure 2a shows that the COFs of specimen No. 1 are gradually increasing during the whole testing period in a distance of 60 m, with a max COF of 0.324, which is the lowest COF of all the laser-quenched specimens but larger than that of the specimen without laser treatment (specimen No. 0). The COF of specimen No. 5 increases quickly in the first 8 s, and then slightly increases to about 0.6, which is the largest COF of all the specimens in Groups I and II (Nos. 0–6). Similarly, Figure 2b shows that specimens No. 9 and 11 have the largest COFs of all the specimens in Groups III and IV (Nos. 7–12), while specimen No. 7 has the lowest COF of all the specimens in Groups III and IV. However, all the laser-quenched specimens have larger COFs than the untreated specimen (No. 0). 

In summary, the COFs of all the laser-surface-quenched specimens except specimen No. 1 increase quickly in the first 8 s, and then increase gradually over the whole testing period. Specimen Nos. 2, 5, 9, and 11 have larger COFs than other laser-quenched specimens, while specimen Nos. 1 and 7 have smaller COFs (0.3 or 0.4) than other laser-quenched specimens in the same group but larger COFs than the untreated cast iron specimen. This finding contradicts previous studies that laser surface treatment can reduce the COFs of laser-modified surfaces [14]. A possible reason is that in this study, a higher laser power, more than 2000 W, was used. As a result, the internal organization of cast iron is densified, the surface hardness is increased, and its wear resistance is enhanced, leading to a higher and more stable coefficient of friction.

### 3.2. Frictional Vibrations and Noise

**Figure 3:**
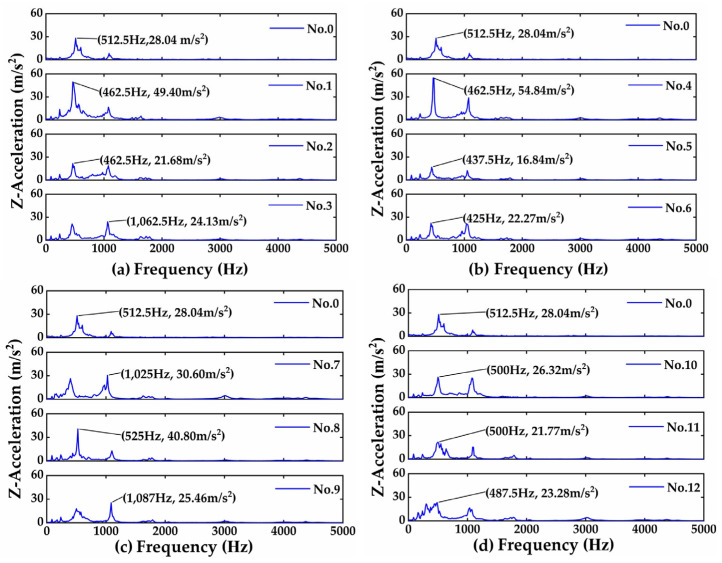
Frequency responses of frictional vibrations with bearing steel balls: (**a**) Nos. 0, 1–3 (Group I); (**b**) Nos. 0, 4–6 (Group II); (**c**) Nos. 0, 7–9 (Group III); (**d**) Nos. 0, 10–12 (Group IV).

Figure 3 shows the frictional vibrations in the frequency domain, as the upper specimens (bearing steel balls) have sliding contact with the lower specimens (cast iron discs) under the normal load of 20 N and sliding speed of 1 m/s. It can be seen from Figure 3 that there are two significant resonant peaks at about 500 Hz and 1050 Hz in all of the tests, which are the natural frequencies of the tribometer. Compared with unprocessed samples, the vibration peaks of laser-treated specimen Nos. 2, 3, 5, 6, and 9–12 are all reduced. Under the same laser quenching power, the vibration acceleration amplitudes of specimen Nos. 2, 5, 9, and 11 are lower compared with other specimens in the same group. It shows that these specimens have better vibration-damping performance in this study.

**Figure 4:**
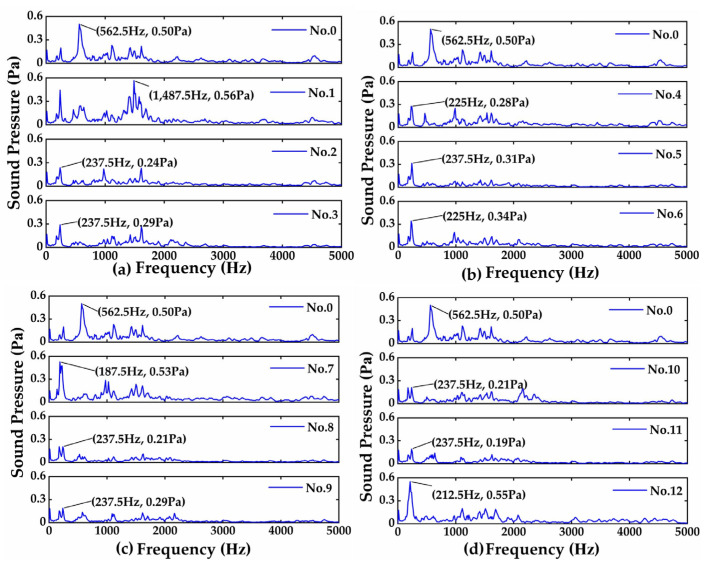
Frequency responses of frictional noise with bearing steel balls: (**a**) Nos. 0, 1–3 (Group I); (**b**) Nos. 0, 4–6 (Group II); (**c**) Nos. 0, 7–9 (Group III); (**d**) Nos. 0, 10–12 (Group IV).

Similarly, Figure 4 presents the measured frictional noise (sound pressure) in the frequency domain from bearing steel balls’ sliding contact with cast iron discs. It is observed from Figure 4 that the peak noise of the untreated specimen occurs at about 562.5 Hz, with a sound pressure of 0.50 Pa. Compared with the unprocessed samples, the sound pressures of samples 2–6 and 8–11 are reduced. Among them, specimen Nos. 9 and 11 have better noise reduction performance than other specimens in this study, in which the sound pressures are reduced by more than 50%.

In order to compare the frictional vibrations and noise more accurately, Table 2 presents the RMS (root mean square) of the amplitudes of frictional vibrations and noise induced by the bearing steel balls’ sliding contact with the cast iron discs. It is observed from Table 2 that the RMS values of frictional vibrations and noise of specimen Nos. 2, 4, 9, and 11 are smaller than that of the untreated specimen, and specimen No. 5 has the most significant reduction in frictional vibrations and noise, i.e., a nearly 40% reduction in frictional vibrations and a 37% reduction in frictional noise, while specimen No. 9 has a 63% reduction in frictional noise and a 9% reduction in frictional vibrations. However, Table 2 also shows that the frictional vibrations and noise from some specimens with improper laser quenching parameters are not reduced but increased, indicating that the laser quenching parameters are critical to the dynamical performance of the laser-quenched material (cast iron), which is required to be further studied in the future.

### 3.3. Hardness

#### 3.3.1. Surface Hardness

**Figure 5:**
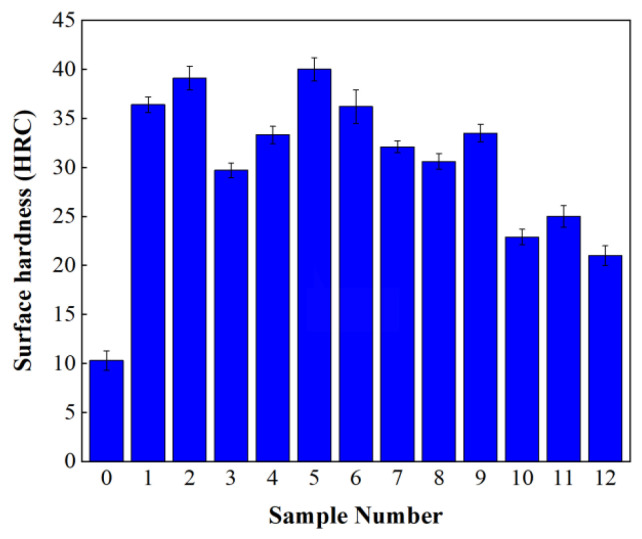
Surface hardness of specimens with and without laser quenching.

In order to check the quality of laser surface quenching, the surface hardness of the laser-quenched cast iron specimens was measured by an HRS-150 Rockwell hardness tester (made by Shanghai Shangcai Testing Machine Co., Ltd., Shanghai, China). Figure 5 shows the average surface hardness of laser-surface-quenched cast iron specimen Nos. 0–12. It is observed from Figure 5 that the hardness of all laser-quenched specimens increased significantly, and the averaged surface hardness of No. 5 is the largest (40.3 HRC), 4 times that of the untreated cast iron specimen (No. 0). The surface hardness of specimen No. 2 (40 HRC) is slightly smaller than that of specimen No. 5. It is also observed that the hardness values of specimen Nos. 2, 5, 9, and 11 are the largest in Groups I, II, III, and IV, respectively. However, the LQI values of specimen Nos. 2, 5, 9, and 11 are not the largest in the same groups, suggesting that the surface hardness of the laser-quenched specimen has a nonlinear relationship with LQI. 

#### 3.3.2. Sub-Surface Micro-Hardness

**Figure 6:**
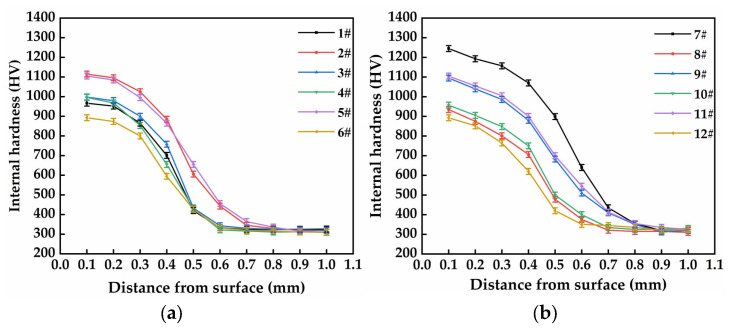
Internal hardness of specimens: (**a**) Nos. 1–6; (**b**) Nos. 7–12.

The sub-surface or internal hardness profiles of the laser-quenched specimens were measured by a digital hardness tester (Shanghai Taiming Co., Ltd., Shanghai, China). Before the sub-surface micro-hardness measurement, the laser-quenched cast iron discs were cut into smaller specimens, and the cross-sectional surfaces of the smaller specimens were polished and ultrasonic-cleaned. In the measurement, a normal load of 3 N was applied and maintained for 15 s. Figure 6 presents the measured sub-surface micro-hardness of the laser-quenched specimens. It is observed from Figure 6a that the micro-hardness values of specimen Nos. 2 and 5 are significantly larger than those of specimen Nos. 1, 3, 4, and 6. Figure 6a also shows that the hardened depth of laser-quenched specimen Nos. 2 and 5 extends to about 0.8 mm, while the laser-hardened depth of specimen Nos. 1, 3, 4, and 6 is about 0.6 mm.

Figure 6b shows that the sub-surface micro-hardness of specimen No. 9 is significantly higher than that of other specimens in this study, and that the sub-surface micro-hardness values of specimen Nos. 7 and 11 are very close and significantly larger than those of specimen Nos. 8, 10, and 12. The hardening depth of specimen Nos. 7, 9, and 11 is about 0.9 mm, while the hardening depth of specimen Nos. 8, 10, and 12 is about 0.7 mm.

### 3.4. Residual Stress

**Figure 7:**
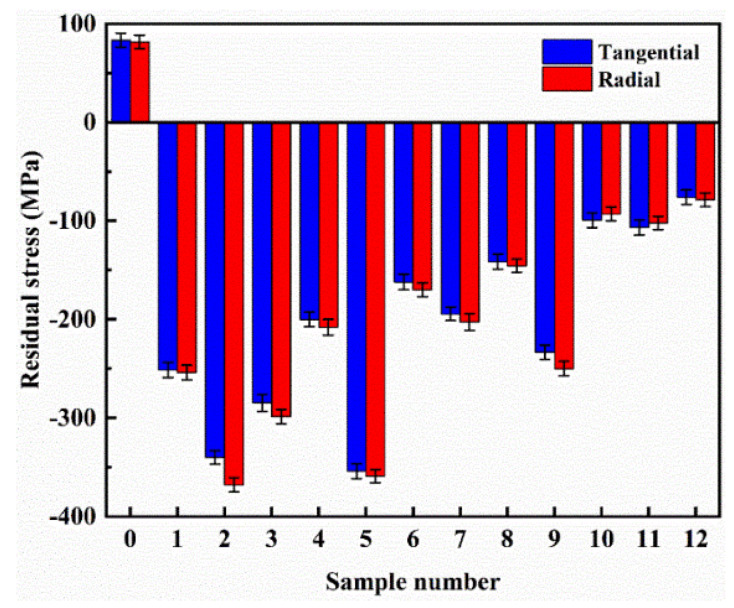
Measured residual stress of cast iron specimen with and without laser quenching.

The TEC-4000 X-ray diffraction system (TEC-Materials Testing, Knoxville, TN, USA) was employed to measure the residual stresses of the specimens in this study. Figure 7 presents the measured mean residual stresses of the untreated and the twelve laser-quenched specimens, showing that the tangential and radial residual stresses on the surfaces of laser-quenched specimens are all compressive but the tangential and radial residual stresses of the untreated specimen are tensile. Both the tangential and radial residual stresses of specimen Nos. 2, 5, and 9 are significantly larger than those in Groups I, II, and III, suggesting that the compressive residual stress of laser-quenched cast iron specimen is correlated with its surface hardness after laser quenching. Considering that specimen Nos. 2, 5, and 9 have better performance in vibration and noise reduction, it is reasonable to conclude that the residual stress or the surface hardness of the specimen may be used as a design parameter for the optimization of the dynamic property (vibration and noise damping) of laser-quenched cast irons. 

This conclusion perhaps cannot be applied to other materials or materials without laser quenching. Normally, if a material has a higher surface hardness, its damping ratio may be smaller, and as a result, the frictional vibration and noise damping will be smaller. However, because the laser quenching changes the surface and internal structures of the material (cast iron in this case) differently and also changes the residual stresses from tensile to compressive stresses, the damping coefficient of the cast iron increases, which improves its dynamic performance. In order to verify this finding, experimental modal testing of the 13 cast iron specimens was carried out, which is described in the following section.

### 3.5. Experimental Modal Testing

**Figure 8:**
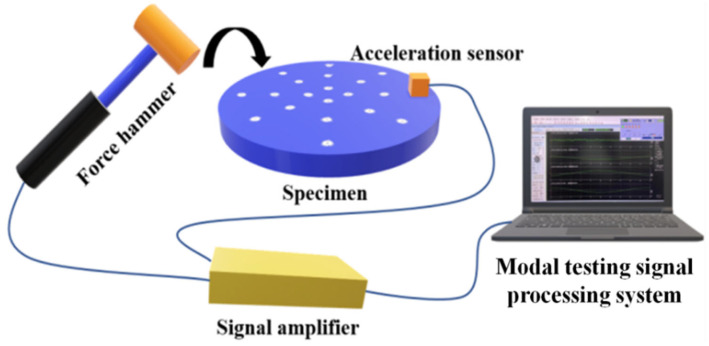
Schematic diagram of modal testing.

In order to investigate the effects of laser quenching on the natural frequencies and damping ratios of the cast iron specimens, standard experimental modal testing was carried out with the DASP (Data Acquisition & Signal Processing) system developed by the BOIVN, Beijing, China. In the modal testing, a hammer with a built-in force sensor (INV931X, BOIVN, Beijing, China) was used to excite the specimen, the responses of the specimen were picked up by an acceleration sensor (INV9832-50, BOIVN, Beijing, China), and the measured signals were processed by the DASP system. Figure 8 illustrates the schematic diagram of modal testing in this study, while Table 3 presents the first three natural frequencies and modal damping ratios of all the specimens with and without laser quenching. 

Table 3 shows that laser surface quenching has no significant effect on the modal frequencies of cast iron specimens, but that the damping ratios of the specimens were increased by laser quenching: the damping ratios of specimen Nos. 2, 5, and 9 were increased significantly. 

Recalling that the compressive residual stresses and the vibration/noise reduction performance of specimen Nos. 2, 5, and 9 were superior to those of the other specimens, it can be concluded that laser quenching with optimal parameters significantly increased the damping ratios and compressive residual stresses of the cast iron specimens, and so significantly improved their vibration and noise damping performance. 

### 3.6. Metallographic Observation

**Figure 9:**
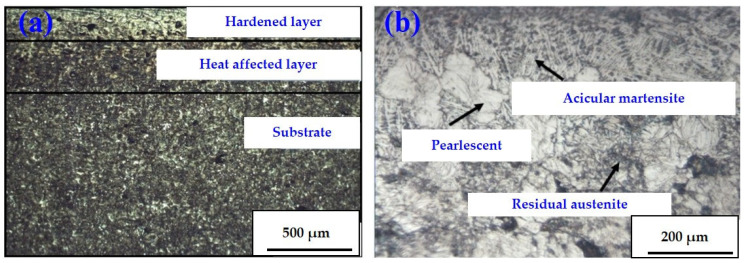
Metallography of specimen No. 5: (**a**) cross-section with 40× magnification; (**b**) hardened layer with 400× magnification.

Figure 9a demonstrates the microstructure of specimen No. 5 observed by the metallographic microscopy with 40× magnification. The cross-sectional metallographic structure of the laser-quenched cast iron specimen can be divided into three layers: a phase hardening layer, a heat-affected layer (transition zone), and a matrix layer. The composition of the phase hardening layer of the specimen is similar to that of traditional quenching, and its structural composition is mainly martensite, carbide, and retained austenite. However, because the heating and cooling rates of laser quenching are faster than traditional quenching, finer and more uniform structures were formed in the hardened layer of the specimen, contributing to the superior mechanical and tribological properties of the laser-hardened structures. 

Figure 9b shows the hardened layer metallographic structure of specimen No. 5 with 400× magnification. It is observed from Figure 9b that the layer near the quenched surface has some pearlite and retained austenite composition in addition to the fine needle martensite. The martensite has a body-centered tetragonal crystal structure, which gives the hardened layer high strength and hardness, good toughness, better wear resistance, and more stable frictional behavior.

**Figure 10:**
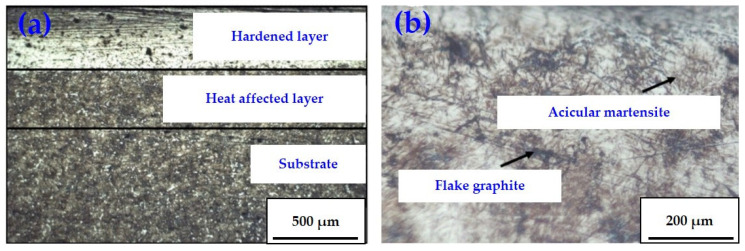
Metallography of specimen No. 9: (**a**) cross-section with 40× magnification; (**b**) hardened layer with 400× magnification.

Figure 10a shows the metallography of specimen No. 9 observed by microscopy with 40× magnification, while Figure 10b demonstrates the structure of the hardened layer of specimen No. 9 under 400× magnification. Compared with other specimens in Group III (the same laser quenching power), the hardened layer of specimen No. 9 is finely organized with a lower impurity content. After laser quenching, the specimen is more fully austenitized. During quenching, most of the austenite was transformed into fine needle-like martensite, and the martensite content in the microstructure of the specimen was increased, which may explain the superior dynamical and tribological properties of specimen No. 9 in Group III. 

### 3.7. Phase Composition

**Figure 11:**
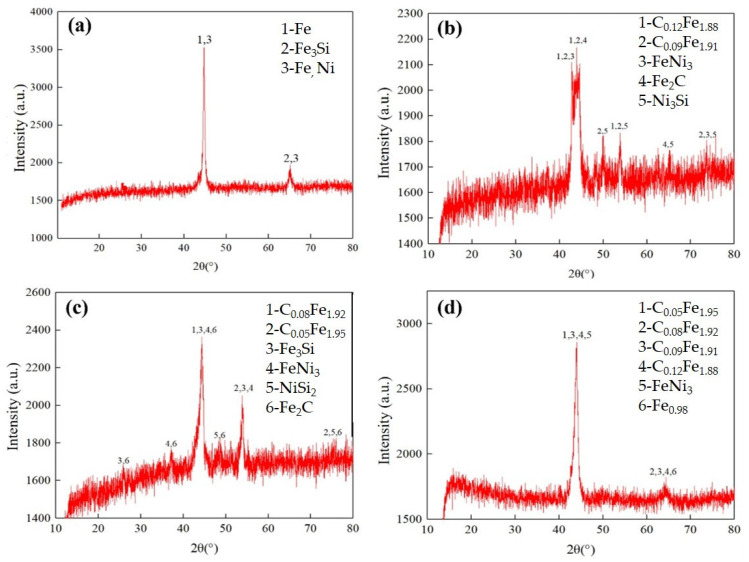
XRD patterns of specimens with and without laser quenching: (**a**) untreated specimen No. 0; (**b**) No. 5; (**c**) No. 9; (**d**) No. 11.

In this study, the untreated specimen No. 0 and specimen Nos. 5, 9, and 11 were selected for the XRD (manufactured by Bruker Technology Co., Ltd., Billerica, MA, USA) analysis, and Figure 11 presents the measured results. It can be seen that the phase composition of specimen No. 0 is mainly Fe, and [Fe, Ni]; the phase composition of specimen No. 5 is mainly C_0.12_Fe_1.88_, C_0.09_Fe_1.91_, FeNi_3_, Fe_2_C, and Ni_3_Si; the phase composition of specimen No. 9 is mainly C_0.08_Fe_1.92_, C_0.05_Fe_1.95_, Fe_3_Si, FeNi_3_, and Fe_2_C; and the phase composition of specimen No. 11 is mainly C_0.05_Fe_1.95_, C_0.09_Fe_1.91_, C_0.12_Fe_1.88_, and FeNi_3_. 

Figure 11 also shows that one diffraction peak is prominent in the XRD spectra of the untreated specimen, but more diffraction peaks are observed in the XRD spectra of specimen Nos. 5, 9, and 11, which means that after laser quenching, some new compounds were developed, and the phase composition changes from a single element or simple compound to complex compounds of C and Fe. The compounds of C and Fe are the main components of martensite obtained after austenitizing the matrix. In the metallographic study, it was observed that the microstructure of the hardened layer is mainly acicular martensite.

In addition, it can be seen that, compared with the untreated specimen, the diffraction peak intensities of specimen Nos. 5, 9, and 11 are decreased by various degrees, indicating that by laser quenching, the single element in the matrix structure decreased but transformed into other compounds of C and Fe. Meanwhile, the half-peak widths of the diffraction peaks of specimen Nos. 5, 9, and 11 (2θ = 45°) increased, which indicates that after laser quenching, the microstructure composition of the specimen becomes refined and the microstructure grain size is decreased, which is consistent with matrix transformation into acicular martensite, as shown in the metallographic observations.

The XRD phase composition analysis supports the metallographic observations, indicating that laser surface quenching changed the phase compositions of the specimens, resulting in an improvement in the dynamical and tribological performance of the laser-quenched specimens.

### 3.8. Electrochemical Analysis

**Figure 12:**
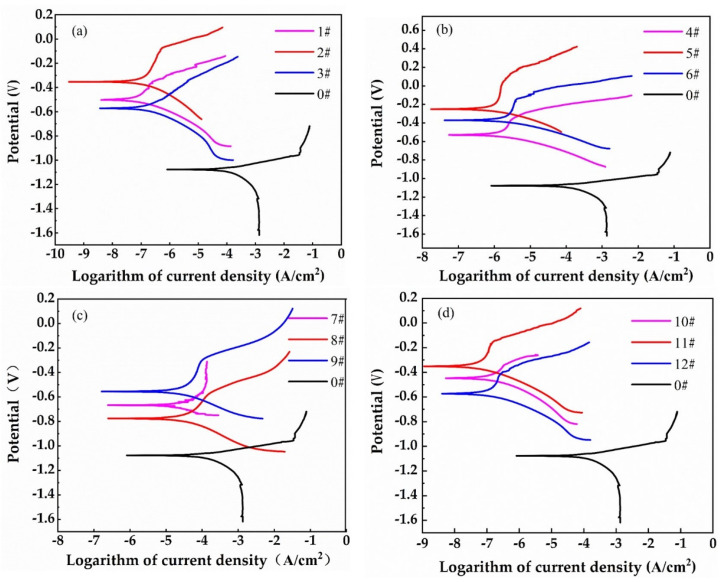
Potentiodynamic polarization curves of specimens: (**a**) Nos. 0–3; (**b**) Nos. 4–6, No. 0; (**c**) Nos. 7–9, No. 0; (**d**) Nos. 10–12, No. 0.

In the electrochemical analysis, the Gamry Electrochemical Workstation (made by the Gamry Electrochemical Instrument Co., Ltd., Warminster, PA, USA) was used to analyze the electrochemical corrosion behavior of cast iron specimens with and without laser quenching. Figure 12a–d presents the potentiodynamic polarization curves of specimen Nos. 0–3; Nos. 0, 4–6; Nos. 0, 7–9; and Nos. 0, 10–12, respectively. This shows that the initial corrosion voltage of the specimen after laser quenching is higher than that of the specimen without laser treatment. In the anode area, the sample processed by laser quenching has a clear active dissolution area, a stable passivation area, and an over-passivation area, while the current density of the specimen without laser treatment increases steadily with the increase in corrosion voltage. For the specimen without laser treatment, there is no obvious stable passivation area because the passivation film on the surface of the untreated cast iron was damaged by Cl^−^ ions in NaCl solution. 

This suggests that the laser-quenched layer of the specimen prevents anions in the electrolyte solution from passing through the specimen surface and chemically reacting with the matrix, thus improving the corrosion resistance of the specimen. When chloride ions in NaCl electrolyte permeate into the specimen, the metal ions in the stable anode will move irregularly, which will reduce the activation energy of the material anode and cause the anode to lose its equilibrium state and become more prone to corrosion. However, after the specimen is laser-quenched, a dense, hardened layer is formed that can reduce the entry of chloride ions to a certain extent, slow down the corrosion of chloride ions to the inside of the specimen, and thus enhance the corrosion resistance of the laser-quenched specimen.

### 3.9. TEM Analysis

**Figure 13:**
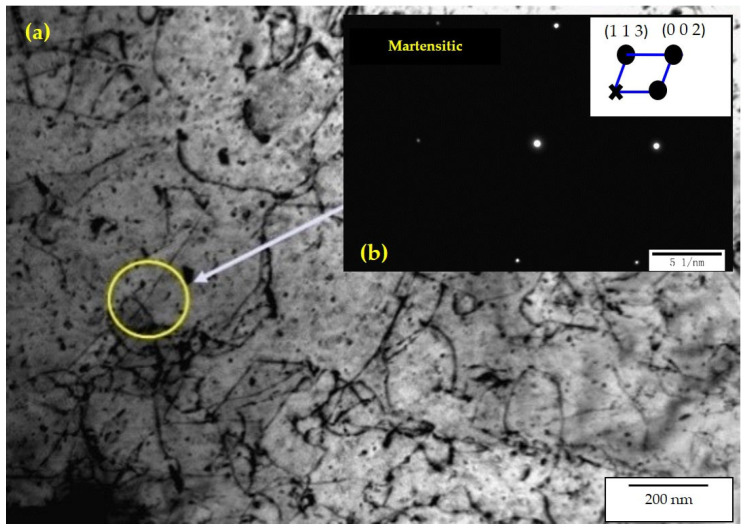
(**a**) Bright-field image of TEM and (**b**) corresponding diffraction pattern of laser-quenched surface of specimen No. 2.

**Figure 14:**
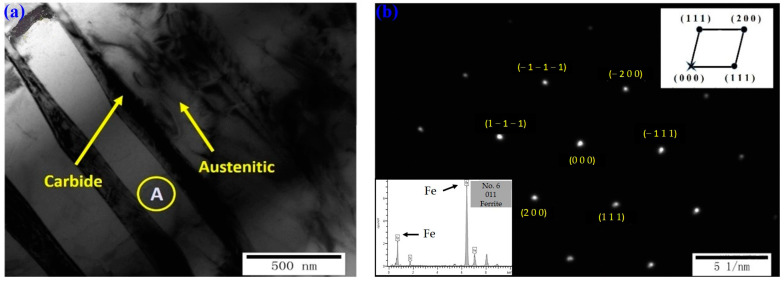
Laser-quenched surface of specimen No. 6: (**a**) bright-field image of TEM, (**b**) corresponding diffraction pattern of area A.

Two specimens with preferred antifriction and wear performance, Nos. 2 and 6, were selected for the TEM (made by JEOL, Showashima, Tokyo, Japan) analysis. Figure 13 shows the bright-field image of transmission electron microscopy and the corresponding diffraction pattern of specimen No. 2; Figure 14 shows the bright-field image of transmission electron microscopy and energy spectrum of specimen No. 6. 

Figure 13 shows that most of the hardened area has been transformed into acicular martensite. The surface hardening zone demonstrates grain refinement due to the rapid cooling rate after laser quenching; most of the crystals grow preferentially along the direction perpendicular to the partition interface, and the branches have a bainitic eutectic structure, interspersed with the retained austenite not transformed during cooling. The grain substructure is finer, the dislocation density is increased, and the thickness of the retained austenite between the grains is greatly reduced, indicating that the extent of residual austenite is significantly reduced. Owing to the high saturation of carbon in the martensite, the hardness of the hardened layer is substantially increased, which explains the excellent performance of specimen No. 2 in the hardness test.

On the other hand, it can be seen from Figure 14 that since the LQI value of specimen No. 6 is smaller than that of specimen No. 2, the hardened zone is mainly martensitic and retained austenite due to the precipitation of carbides from the supersaturated α-solid-solution of the original martensite as a result of the natural cooling temperature. In the immediate vicinity of the precipitated carbide, martensite carbon, chromium concentration is sharply reduced to form a chromium-poor area, so the metallographic organization of the carbonized area is susceptible to corrosion and a darker color. 

## 4. Conclusions

In this study, the effects of high-power laser quenching on the surface and sub-surface properties of cast iron, including vibration/noise reduction, friction/wear performance, corrosion resistance, and hardening mechanism, were investigated by means of dynamical and tribological tests, residual stress measurements, electrochemical analysis, surface and sub-surface micro-hardness testing, metallographic observations, and XRD and TEM analyses. The conclusions can be drawn as follows.

(1) The surface hardness of the laser-quenched specimens was increased by up to 4 times, the sub-surface micro-hardness of the laser-quenched specimens was increased significantly, and the depth of the hardened layer was in the range of 0.6–0.9 mm. Specimen Nos. 2, 5, 9, and 11 have larger surface and sub-surface hardness than others in the same group.
(2) Laser quenching significantly increased the residual stress on the surface of cast iron specimens and changed it from tensile to compressive. In the same group of laser-quenched specimens, the compressive residual stresses of specimen Nos. 2, 5, 9, and 11 increased more markedly than those of the other specimens.
(3) Specimen Nos. 2, 5, 9, and 11 have better vibration and noise reduction performances than those of other specimens in the same group due to increased damping and the presence of larger compressive residual stresses generated by laser quenching, although the surface hardness and COFs of these specimens against bearing steel are larger, suggesting that the compressive residual stress and damping coefficient of a functional material (cast iron) are more critical to its dynamical performance than surface hardness and COF.
(4) Through metallographic, XRD, and TEM observations, it was found that the microstructures of cast iron specimens significantly changed after laser quenching. Most of the hardened area has been transformed into acicular martensite and it has microstructures due to grain refinement and recrystallization. Due to the high saturation of carbon in martensite, the wear and corrosion resistance of the laser-quenched layer was significantly improved.

## Figures and Tables

**Table 1:** LQI values used for the laser quenching of the cast iron specimen ($GW/(m^{3}\cdot s)$).

No.	1	2	3	4	5	6	7	8	9	10	11	12
LQI	12.0	10.2	9.0	10.8	9.0	8.4	13.8	12.0	10.2	7.5	6.6	7.5

**Table 2:** RMS of frictional vibrations and noise from bearing steel balls’ contact with cast iron specimens.

No.	LQI	Peak Vibration Amplitude (m/s^2^)	RMS (m/s^2^)	Ratio	Peak Sound Pressure (Pa)	RMS (Pa)	Ratio
0	/	28.04	1.245	1.00	0.50	0.020	1.00
1	12.0	49.40	2.082	1.77	0.56	0.032	1.12
2	10.2	21.68	1.067	0.77	0.24	0.017	0.47
3	9.0	24.13	1.132	0.86	0.29	0.017	0.58
4	10.8	54.84	1.721	1.96	0.28	0.022	0.55
5	9.0	16.84	0.755	0.60	0.31	0.011	0.63
6	8.4	22.27	1.120	0.79	0.34	0.016	0.68
7	13.8	30.60	1.435	1.09	0.53	0.030	1.05
8	12.0	40.80	1.061	1.45	0.21	0.012	0.41
9	10.2	25.46	1.030	0.91	0.19	0.012	0.37
10	7.5	26.32	1.358	0.94	0.21	0.017	0.42
11	6.6	21.77	1.105	0.78	0.19	0.012	0.38
12	7.5	23.28	1.552	0.83	0.55	0.021	1.10

**Table 3:** Experimental measured modal parameters of specimens.

Specimen No.	Measured Modal Frequencies (kHz)			Measured Modal Damping Ratios (%)		
	First	Second	Third	First	Second	Third
0	8.4	10.9	18.5	1.78	0.14	0.13
1	8.1	10.0	18.3	3.00	1.15	0.22
2	7.9	10.6	18.0	5.43	2.18	0.89
3	8.3	11.7	18.4	4.33	1.38	0.43
4	7.8	12.1	18.0	2.37	1.44	0.23
5	7.7	12.8	18.5	5.38	2.41	0.46
6	7.7	12.9	18.5	2.84	1.02	0.31
7	8.3	12.8	18.4	2.60	1.25	0.60
8	7.6	12.9	18.4	2.61	1.50	0.48
9	7.8	12.9	18.3	3.52	1.98	0.63
10	8.3	12.9	18.2	2.16	1.07	0.20
11	8.4	12.8	18.1	3.78	1.10	0.48
12	8.4	12.9	18.3	2.83	1.15	0.23

## Data Availability

Not applicable.
